# Curcumin Enhances Radiosensitization of Nasopharyngeal Carcinoma via Mediating Regulation of Tumor Stem-like Cells by a CircRNA Network

**DOI:** 10.7150/jca.39511

**Published:** 2020-02-10

**Authors:** Daoqi Zhu, Meng Shao, Jiabin Yang, Miao Fang, Shiya Liu, Dandan Lou, Ruijiao Gao, Ying Liu, Aiwu Li, Ying Lv, Zhixian Mo, Qin Fan

**Affiliations:** 1 School of Traditional Chinese Medicine, Southern Medical University, Guangdong Guangzhou, 510515, China; 2 NanFang Hospital, Guangdong Guangzhou, 510515, China

**Keywords:** nasopharyngeal carcinoma, circRNA, tumor stem-like cells, radiosensitization, curcumin

## Abstract

Circular RNAs (circRNAs) are involved in cancer development via inhibition of miRNAs, which are associated with differentiation, proliferation, migration, and carcinogenicity. Curcumin has antioxidant and anti-cancer properties, and it has also been used as a radiosensitizer. In this study, we explored the potential relationships among curcumin, circRNAs, and nasopharyngeal carcinoma (NPC). We compared the differences in circRNA levels in NPC cell lines after radiotherapy and after treatment with curcumin, using a high-throughput microarray. Further, a circRNA-miRNA-mRNA interaction network between radiation resistance NPC cell lines and tumor stem cells was constructed by applying bioinformatics. Finally, it was demonstrated by reverse transcription-quantitative polymerase chain reaction assay and wound healing assay that curcumin could enhance radiosensitization of NPC cell lines via mediating regulation of tumor stem-like cells by the "hsa_circRNA_102115"-"hsa-miR-335-3p"-"MAPK1" interaction network.

## Introduction

Nasopharyngeal carcinoma (NPC) is an endemic tumor in certain parts of Southeast Asia, especially China (Canton) and Hong Kong 1. Epstein-Barr virus (EBV) infection, genetic, environmental, and dietary factors contribute to the development of NPC 2. Few genetic signatures have been previously identified for the early diagnosis and long-term risk prognosis of NPC 3, 4. For the past decades, chemotherapy combined with radiotherapy is the first therapeutic option in loco-regionally advanced NPC 5. With treatment, the rates of locoregional relapse, distant metastases, and five-year survival are 16%, 31.5%, and 71.4%, respectively, which can be considered as satisfactory 3. Accumulating evidence has substantiated that cancer stem cells (CSCs) or tumor side populations (SP) have self-renewal and stem-like cell properties, and play a crucial role in tumor resistance and relapse to radiotherapy 6. Cancer stem cells are related to tumor metastasis and relapse after standard treatment in NPC 7.

Curcumin (CUR), a natural bioactive compound extracted from a traditional Chinese medicinal herb turmeric (*Curcuma longa*), has been reported to exhibit potent antitumor activities against different types of cancer, including NPC in both *in vivo and in vitro* models 8. Our previous studies have shown that curcumin could enhance radiosensitization in NPC by variant pathways through the regulation of different coding- or noncoding-RNAs 9. Current studies have shown that curcumin exerts prominent inhibitory effects on various cancer stem cells through different pathways. Curcumin attenuates the malignant potential of glioblastoma stem cells by induction of reactive oxygen species (ROS) and activation of the mitogen-activated protein kinase (MAPK) pathway 10. Curcumin suppresses metastasis in colorectal cancer (CRC) by modulating crosstalk between colon cancer stem cells and stromal fibroblasts in the tumor microenvironment, at least in part by the mediation of transforming growth factor-β (TGF-β) and epithelial-mesenchymal transition (EMT) 11. Curcumin also enhances the effects of cisplatin by targeting the CSCs CD166+/EpCAM+ subpopulation in non-small cell lung cancer cell lines (NSCLC) by p21- and cyclin D1-mediated tumor cell inhibition 12. Further, curcumin inhibits EMT and migration of breast CSCs by promoting the E-cadherin/β-catenin negative feedback loop 13. Curcumin is a potential drug to augment the radiosensitivity of NPC by inhibiting the proliferation and differentiation of NPC stem-like cells.

Noncoding RNAs (ncRNA) play critical roles in the regulation of cellular mechanisms and are classified as microRNAs (miRNA), long non-coding RNAs (lncRNA), and circular RNAs (circRNA), making up 95% of the total RNA in the eukaryotic transcription 14. The competing endogenous RNA (ceRNA) mechanism suggests that lncRNAs and circRNAs serve as natural miRNA sponges by competitively binding to miRNA response elements (MREs) and suppressing their expression and function 15. Accumulating evidence suggests that circRNAs also exert the same effects. CircRNA_100290 was found to inhibit the proliferation of oral cancer *in vitro* and *in vivo* by regulation of CDK6 expression and sponging up miR-29b family members 16. CircRNA-000911 was found to suppress proliferation, migration, and invasion of breast cancer cells and promoted apoptosis via miR‑449a, Notch1, and the nuclear factor-κB (NF-κB) pathway 17. The inhibition of proliferation and invasion of cervical cancer by the miR-506/Snail-2 pathway has also been reported after the silencing of circRNA-000284 18. Meanwhile, very few studies have demonstrated that circRNAs play a significant role in regulating the proliferation and differentiation of CSCs or SP. It was reported that circVRK1 could suppress the expansion and self-renewal capacity of breast CSCs via the circRNA/ miRNA network 19. But just a very few published papers have shown the correlation between circRNAs and NPC, especially radiosensitization or CSCs related. As described by Ke Z, they demonstrated circHIPK3 facilitated NPC progression through protecting ELF3 from miR-4288-mediated silencing, which suggested that the circHIPK3-miR-4288-ELF3 regulatory loop might be a potential target for NPC prevention 20.

Most of the NPC patients are initially diagnosed with undifferentiated and non-keratinizing carcinoma 21. Studies have shown that circRNAs are abundantly expressed in epidermal stem cells (EpSCs), and the expression markedly changes in an integrated manner during the differentiation of the cancer cells 22. Atkinson et al. (2017) suggested that circRNAs take part in the cellular irradiation response by demonstrating KIRKOS-71 and KIRKOS-73 as potential diagnostic biomarkers of radiotherapy 23. Interestingly, with further exposure to radiation, there was marked attenuation of adherence leading to enhanced radio-resistance and formation of a stable radioresistant NPC cell line. Based on the findings of these studies, we hypothesized that continuous stimulation or injury of nasopharyngeal epithelial lesions induce abnormal expression of circRNAs leading to the differentiation of nasopharyngeal epithelial stem cells along with an attempt of self-renewal to replace the impaired cells. Unfortunately, a vast majority of mutations lead to carcinogenesis and the formation of highly radioresistant NPC stem-like cells. Therefore, it is essential to explore the potential relationship among curcumin, circRNAs, and NPC or NPC stem-like cells.

## Materials and Methods

### Cell culture and reagents

The human NPC cell line CNE-2 was obtained from Sun Yat-sen University (Guangzhou, China). The cells were cultured in RPMI-1640 medium (Thermo Fisher Scientific, Inc., Waltham, MA, USA) supplemented with 10% fetal bovine serum (Thermo Fisher Scientific, Inc.) at 37 °C in a humid incubator in the presence of 5% CO_2_. Curcumin was dissolved in DMSO (Sigma-Aldrich, USA) and filtered through a 0.22-μm filter (Millipore, Merck KGaA, Darmstadt, Germany). The curcumin solution was diluted in fresh broth to a concentration of 20 μmol/L before use 24. The cells were divided into three groups: CNE-2 group (NC), IR (irradiation) group (IM), and IR + 20 μmol/L CUR group (IC).

### Microarray hybridization of circRNA

Total RNA was extracted using TRIzol reagent (Invitrogen) and quantified using NanoDrop ND-1000 (Thermo Fisher Scientific, Inc.). The sample preparation and microarray hybridization were performed according to a standard protocol (Arraystar, MD, USA). Briefly, total RNAs were digested with RNase R (Epicentre, Inc., Wisconsin, USA ) to remove the linear RNAs and enrich the circular RNAs. The enriched circular RNAs were amplified and transcribed into fluorescent cRNA utilizing a random priming method (Arraystar Super RNA Labeling Kit, Arraystar). The labeled cRNAs were hybridized onto human circRNA array V2 (8x15K, Arraystar). After washing the slides, the arrays were scanned by Agilent Scanner G2505C (Agilent Technologies, USA).

Agilent Feature Extraction software (version 11.0.1.1, Agilent Technologies) was used to analyze the acquired array images. Quantile normalization and subsequent data processing were performed using R software (limma package). Differentially expressed circRNAs with a significant difference between the two groups were identified by volcano plots. The differentially expressed circRNAs in the two samples were identified through filtering based on fold change. Hierarchical clustering was performed to detect the discernible circRNAs expression pattern in the samples.

### Bioinformatical analysis of the circRNAs/miRNAs/mRNA network

Hierarchical clustering is the primary statistical method for finding the relatively homogeneous clusters of cases based on measured characteristics in large datasets 25. Cluster and TreeView programs were used to reveal the hidden structures of the circRNA expression in the two groups.

The circRNA/miRNA network was predicted by miRNA target prediction software (Arraystar) based on MiRanda and TargetScan and was constructed by Cytoscape 3.01 26. The target mRNAs of the miRNAs were surveyed by DIANA-miRPath (p < 0.05, MicroT < 0.8) and the intergenic interaction network constructed based on the KEGG pathway database 27. The molecular function, biological process, cellular component, protein domain, site of expression, and Catalogue of Somatic Mutations in Cancer (COSMIC) of target miRNAs were analyzed by FunRich (v 3.1.3) (p < 0.05) 28.

### Wound healing assay

A single-cell suspension of CNE-2 was seeded in 6-well plates and cultured until full confluence (cells for IC were exposed to 20 μmol/L CUR for 48 h in the beginning). A straight wound was induced by scratching with a sterile pipette tip which was washed twice with phosphate-buffered saline (PBS). The gap was observed and photographed under a microscope at 200X magnification.

### Reverse transcription-quantitative polymerase chain reaction (qRT-PCR) analysis

Total RNA was extracted using TRIzol reagent (Thermo Fisher Scientific, Inc.) according to the manufacturer's instructions. The expression of mRNA and miRNA were determined by using SYBR Green Master Mix (Thermo Fisher Scientific, Inc.) and StepOne Plus quantitative PCR system (Thermo Fisher Scientific, Inc.) following the manufacturer's instructions. U6 and GAPDH were used as an internal control for miRNA and mRNA, respectively. The fold changes were calculated according to the 2-△△Ct equation. The list of all the primers enumerated in Supplementary Table S1.

### Clonogenic survival assay

CNE-2 cells were selected after irradiation used for the IM group. Different cells were counted and seeded at different cell densities in triplicates in six-well cell culture plates. Nonirradiated and irradiated cells were grown for 7 to 14 days, allowing the surviving cells to produce macroscopic colonies, each consisting of 50 or more cells and stained with Giemsa solution (1:999 with distilled water). The surviving fractions (SF) were calculated by dividing the PE by the PE of the non-irradiated control. Sensitization enhancement ratio was calculated by the division of SF2.

### Western Blots (WB) Analysis of Selected Proteins Expression

Cells were harvested and homogenized in the lysis buffer on ice using the proteo JET mammalian cell lysis reagent (Fermentas Life sciences, Israel). Protein concentration was determined using the Bio-Rad kit (Bio-Rad, Hercules, CA). The membranes with selected proteins were incubated at 4°C overnight with primary antibody against IGF1R (Cat #PA5-85986, Thermo Fisher Scientific, Inc.), MAPK1 (Cat #13-8600, Thermo Fisher Scientific, Inc.), MAPK3 (Cat #PA5-29636, Thermo Fisher Scientific, Inc.) and β-actin(Cat #MA5-15739, Thermo Fisher Scientific, Inc.), and then with the corresponding secondary antibody for 1h at room temperature. Immunofluorescence signals were detected using the ECL kit (Cell Signaling Technology, Inc.) and quantified using on the Gel Logic 2200 PRO Imaging System (Kodak, Rochester, NY, USA).

### Flow cytometry (FCM) Analysis of NPC stem-like cells

CD133 can serve as a specific surface marker for nasopharyngeal cancer stem cells^29^. Cells were harvested and counted, to a volume of 100 μL cell suspension, 5 μL CD133 antibody (Cat # 17-1338-42, Thermo Fisher Scientific, Inc.) mix was added, mixed and incubated for 20 minutes at 4°C in the dark and washed twice with 250 μL DPBS or FACS buffer. The stained cells were analyzed with fluorescence-activated cell sorting (FACS) by FCM (FACS Calibur, Becton Dickinson, Bedford, MA).

### Statistical analyses

All statistical analyses were performed using SPSS software (v 21.0) for Windows (SPSS Inc., Chicago, IL, USA). The continuous data are represented as the mean ± standard deviation. One-way ANOVA was performed, and a p-value of < 0.05 was considered as statistically significant.

## Results

### Changes in expression of circRNAs analyzed by microarray

**Figure 1:**
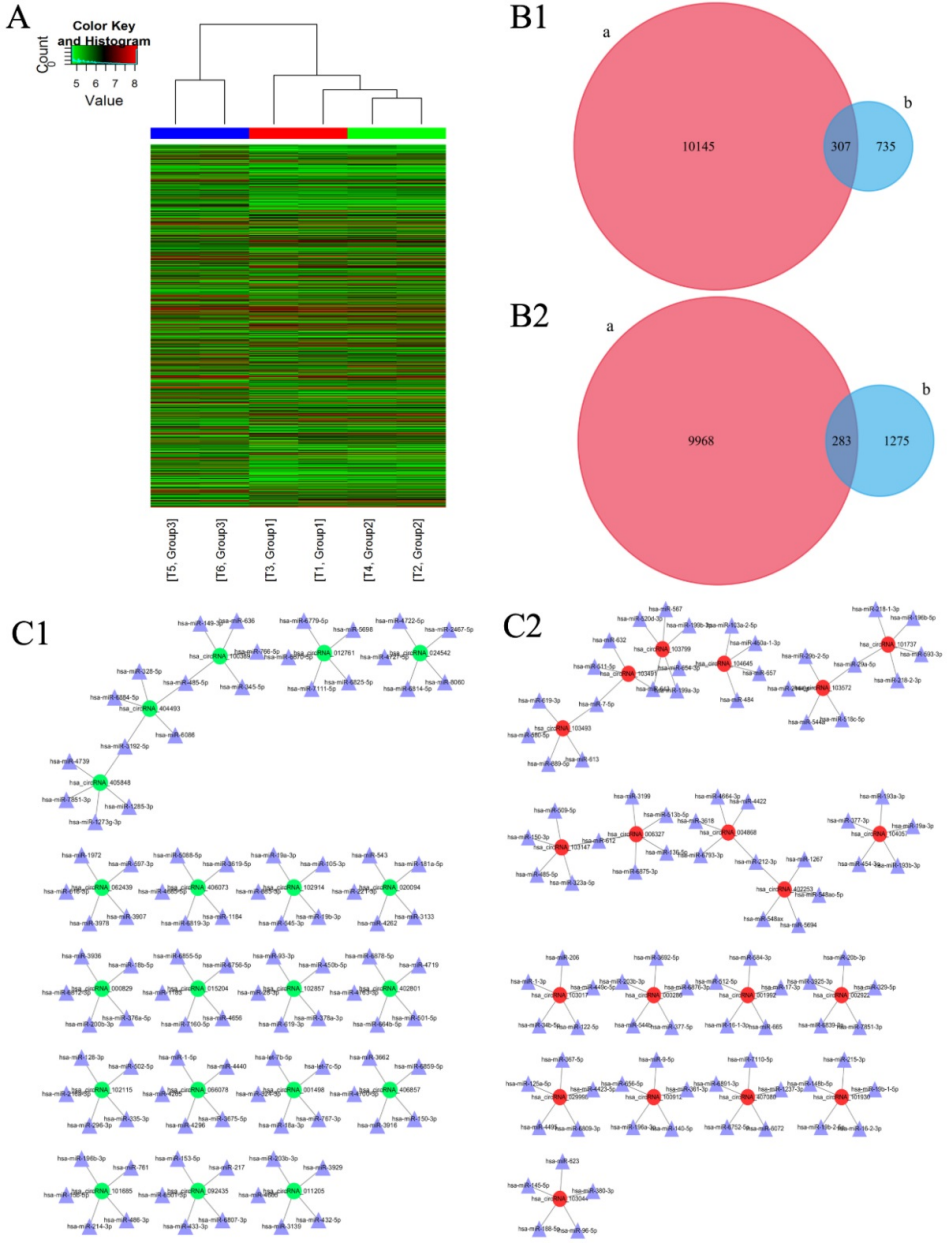
Changes in the expression of circRNAs and the corresponding miRNAs as analyzed by microarray. (A) Hierarchical clustering heatmap showing the expression of all the target circRNAs and the most upregulated and downregulated circRNAs in the three groups. (B1) 307 upregulated-restored circRNAs after exposure to curcumin. (B2) 283 downregulated-restored circRNAs after exposure to curcumin. (C1) The top 20 upregulated-restored circRNAs and the corresponding miRNAs. (C2) The top 20 downregulated-restored circRNAs and the corresponding miRNAs.

One thousand and forty-two circRNAs were significantly upregulated [fold change (FC) ≥ 2.0, p < 0.05] and 1558 circRNAs were significantly down-regulated [FC ≤ 0.5, p < 0.05] in IM group as compared to those in NC group [Figure 1A]. Among these, the expression of 307 circRNAs which were significantly up-regulated and 283 circRNAs which were significantly down-regulated were restored to non-significant levels after CUR intervention [Figure 1B 1-2]. Ninety-one circRNAs were significantly upregulated [FC ≥ 2.0, p < 0.05] and 11 circRNAs were significantly downregulated [FC ≤ 0.5, p < 0.05] in IC group as compared to those in IM group [Figure 1A]. Further, 1486 circRNAs were significantly upregulated [FC ≥ 2.0, p < 0.05] and 1687 circRNAs were significantly downregulated [FC ≤ 0.5, p < 0.05] in IC group as compared to those in NC group [Figure 1A]. The expression of circRNAS in the IM group was upregulated as compared to that in the NC group. The network diagram revealed that after CUR intervenetion, the IC group expressed the most significant reduction in the expression of 20 circRNAs and their corresponding 100 miRNAs as compared to the IM group [Figure 1C 1]. The same with the expression of circRNAs were decreased too [Figure 1C 2].

### Gene ontology (GO) analysis of miRNAs

**Figure 2:**
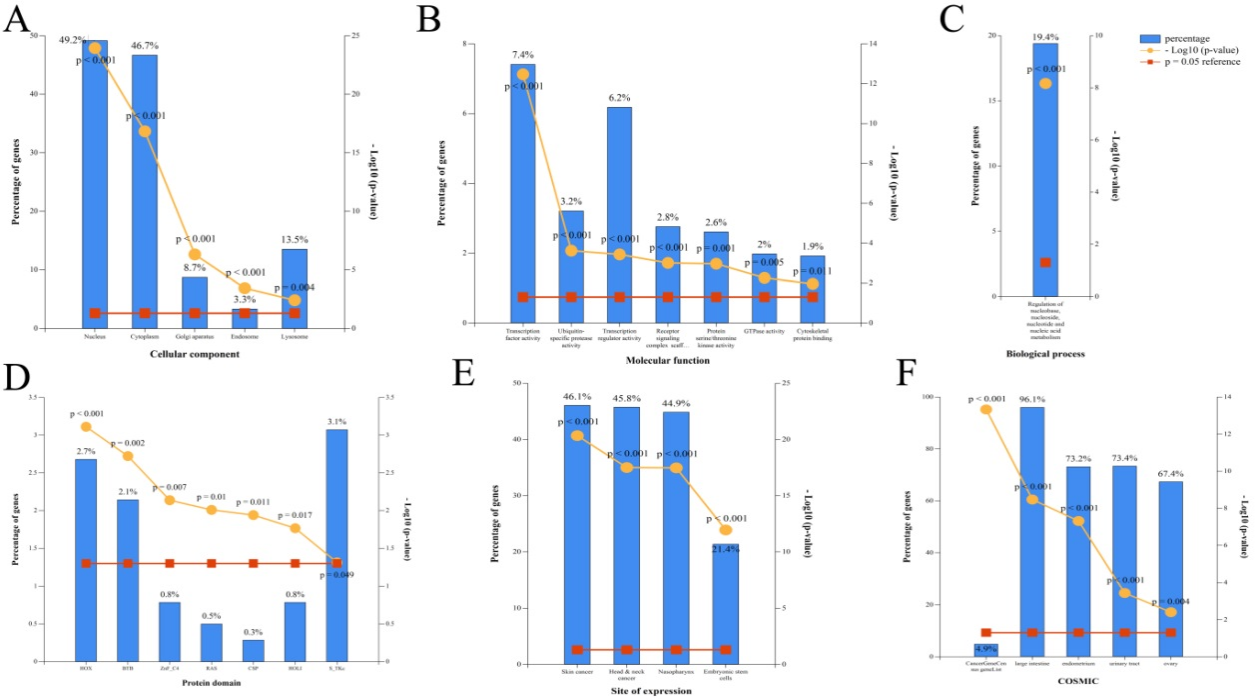
Corresponding miRNA enrichment by GO analysis. (A) Cellular component. (B) Molecular function. (C) Biological process. (D) Protein domain. (E) Site of expression. (F) Catalogue of Somatic Mutations in Cancer (COSMIC).

According to the restoration of expression of the top 20 circRNAs after CUR intervention (ranked by fold change), GO enrichment analysis of the corresponding 100 miRNAs was performed by FunRich (p < 0.05). The cellular component included GO terms nucleus, cytoplasm, Golgi apparatus, endosome, and lysosome [Figure 2A]. The molecular function included GO terms transcription factor activity, ubiquitin-specific protease activity, transcription regulator activity, receptor signaling complex scaffold activity, protein serine/threonine kinase activity, GTPase activity, and cytoskeletal protein binding [Figure 2B]. The biological process included GO terms regulation of nucleobase, nucleoside, nucleotide, and nucleic acid metabolism [Figure 2C]. The protein domain included GO terms HOX, BTB, ZNf-C4, RAS, CSP, HOLI, and S-TKc [Figure 2D]. The site of expression included GO terms of skin cancer, head and neck cancer, NPC, and embryonic stem cells (partly) [Figure 2E]. The catalog of somatic mutations in cancer (COSMIC) included GO terms cancer gene census gene list, large intestine, endometrium, and liver [Figure 2F].

### The circRNA-miRNA-mRNA interaction network

**Figure 3:**
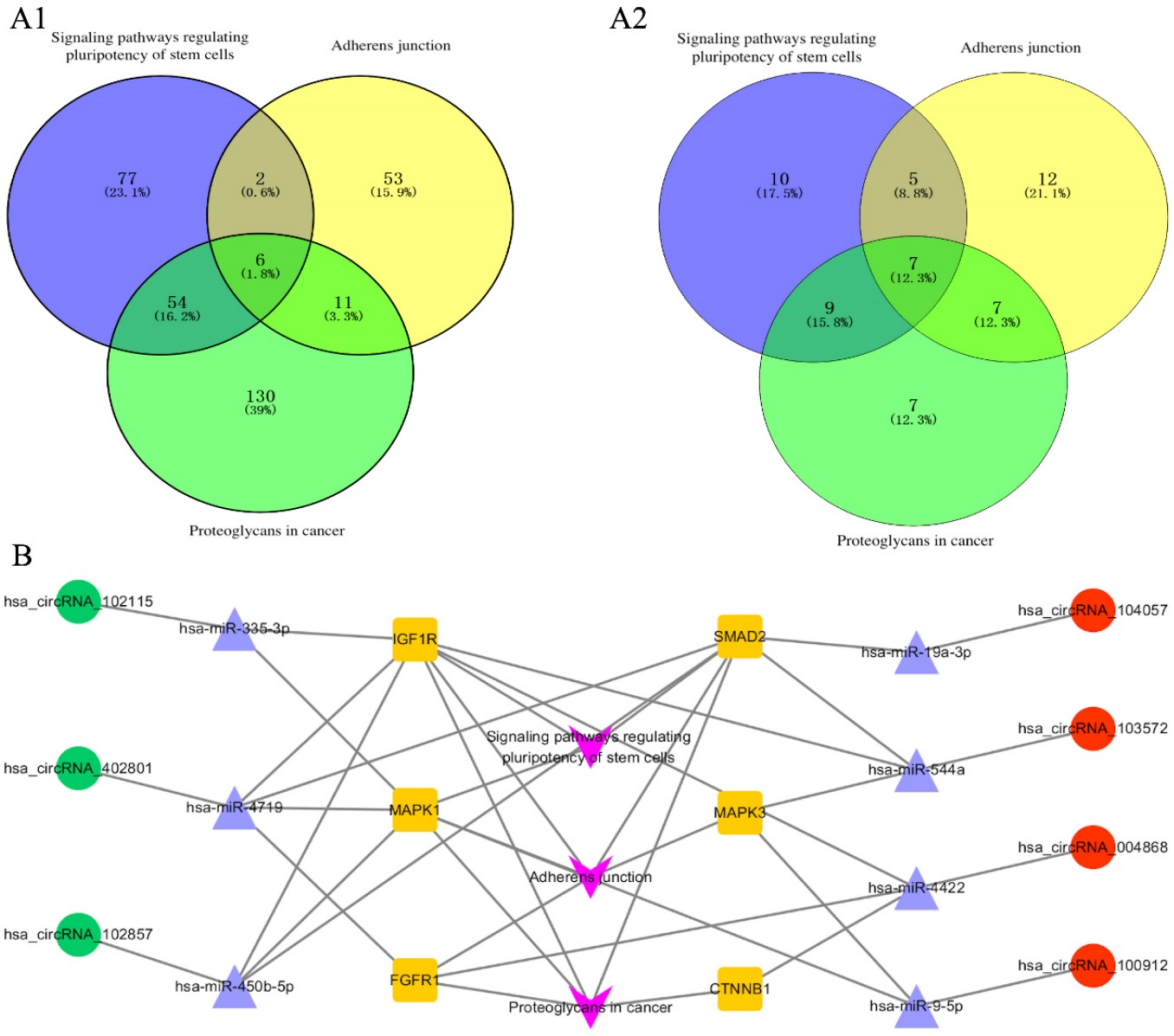
Pathway analyzed with DIANA-mirpath and circRNA-miRNA-mRNA interaction networks. (A1) 6 genes were involved in the three pathways simultaneously. Proteoglycans, adherens junction, and signaling pathways regulating pluripotency of stem cells were analyzed with DIANA-mirpath. (A2) 7 miRNAs were simultaneously involved in the three pathways from the 100 miRNAs including the top 20 miRNAs (ranked by fold change) which restored the expression of circRNAs. (B) The circRNA-miRNA-mRNA interaction network involving the three pathways, 6 genes, 7 circRNAs, and 7 corresponding miRNAs.

The corresponding 100 miRNA analysis was performed using DIANA-mirpath. Proteoglycans in cancer, adherens junction, and signaling pathways regulating pluripotency of stem cells were chosen as the main pathways for further study. Six genes are simultaneously involved in the above three pathways, namely, CTNNB1, FGFR1, IGF1R, SMAD2, MAPK1, and MAPK3 [Figure 3A-1]. Subsequently, the 6 genes were used to find the corresponding miRNAs among the 100 miRNAs. Seven miRNAs were found to be simultaneously involved in the above three pathways, namely, hsa-miR-335-3p, hsa-miR-19a-3p, hsa-miR-544a, hsa-miR-4422, hsa-miR-9-5p, hsa-miR-450b-5p, and hsa-miR-4719 [Figure 3A-2]. The 7 miRNA were used to find the corresponding circRNA among the top 20 circRNAs, hsa_circRNA_102115, hsa_circRNA_104057, hsa_circRNA_103572, hsa_circRNA_004868, hsa_circRNA_100912, hsa_circRNA_102857, and hsa_circRNA_402801. Finally, the 7 circRNA-related miRNAs and mRNA interaction network was constructed [Figure 3B].

### Verification of the circRNA-miRNA-mRNA interaction network

**Figure 4:**
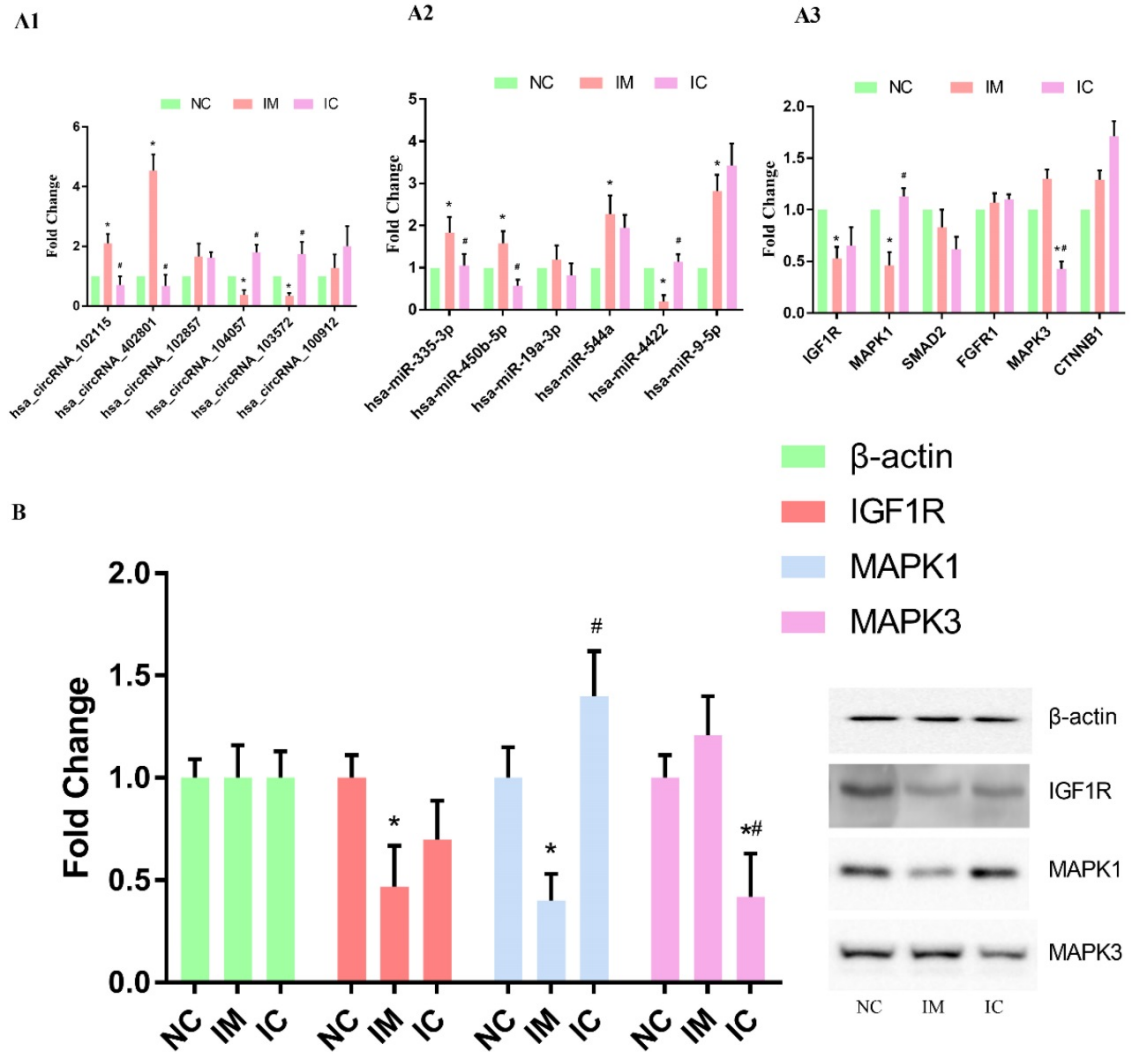
Verification of the circRNA-miRNA-mRNA interaction network. (A1) qRT-PCR detection of circRNA expression in the three groups. (A2) Levels of target miRNAs for circRNA in the three groups. (A3) Levels of target mRNAs for miRNAs in the three groups. The expression of each RNA was calculated using 2-△△Ct equation. Compared to NC group: *p < 0.05, and compared to IM group: #p < 0.05. (B) WB analysis of selected protein expression. Compared to NC group: *p < 0.05, and compared to IM group: #p < 0.05.

We used qRT-PCR to verify the changing trends in the expression of the selected circRNAs and the corresponding miRNAs and mRNAs. The expression of hsa_circRNA_102115, hsa_circRNA_104057, hsa_circRNA_103572, and hsa_circRNA_402801 were found to be changed dramatically in both IM and IC groups [p < 0.05] [Figure 4 A1]. The expression of hsa-miR-335-3p, hsa-miR-544a, hsa-miR-4422, hsa-miR-9-5p, hsa-miR-450b-5p were found to differ significantly in the IM group as compared to those in NC group. Expression of hsa-miR-335-3p, hsa-miR-450b-5p, and hsa-miR-4422, however, were restored significantly [p < 0.05] [Figure 4 A2]. On the other hand, the expression of IGF1R and MAPK1 was found to be changed dramatically in IM group as compared to those in NC group, and expression of MAPK1 was restored significantly [p < 0.05] [Figure 4 A3]. Also, the same results of WB can verify this interaction network in a protein level, the expression of IGF1R and MAPK1 in protein level is significantly lower in IM cells [p < 0.05]. And compared to those in NC group, and expression of MAPK1 was restored significantly [p < 0.05] [Figure 4 B].

### Detection of cell biology changes

**Figure 5:**
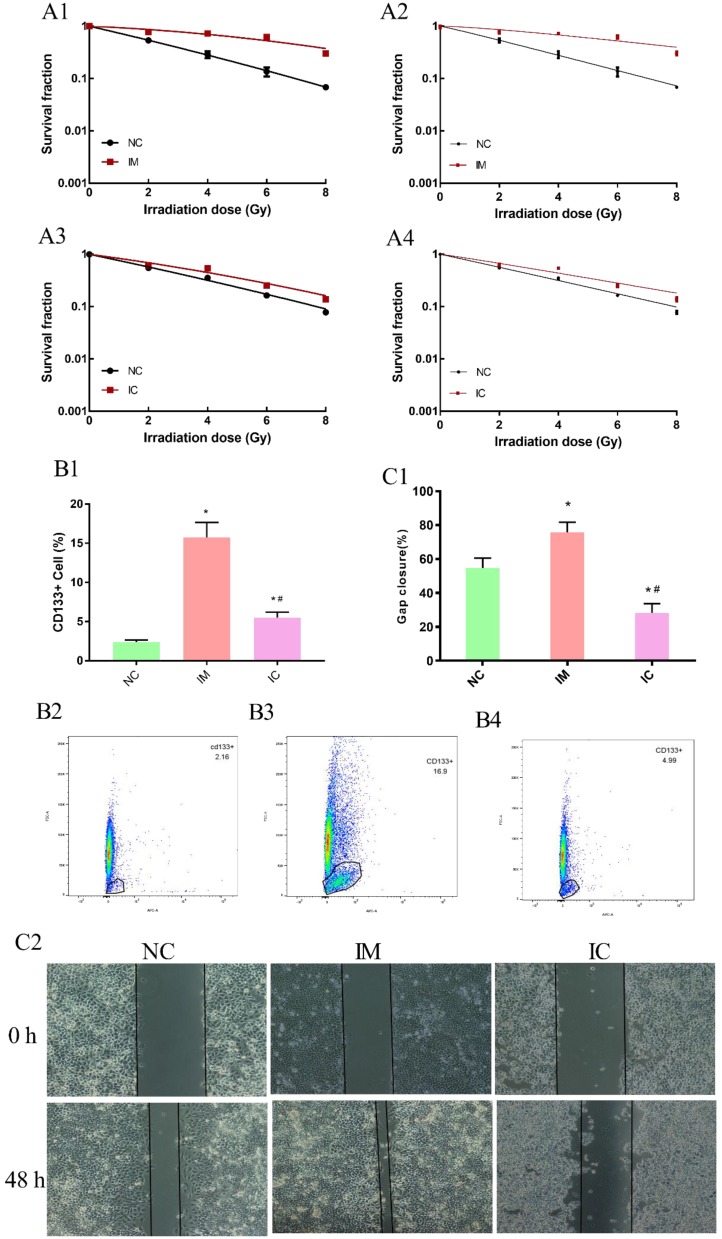
Detection of cell biology changes. The dose-survival curve of NC and IM obtained from L-Q matching. (A1) The dose-survival curve of NC and IM obtained from multi-target single-hit model matching. (A2) The dose-survival curve of NC and IC obtained from L-Q matching. (A3) The dose-survival curve of NC and IC obtained from multi-target single-hit model matching. (A4) The amount of CD133+ cells in three groups. (B1-4) Compared to NC group: ^*^p < 0.05, and compared to IM group: ^#^p < 0.05. Wound healing assay among the three groups. (C2) Gap closure (%) in each group. Compared to NC group: ^*^p < 0.05, and compared to IM group: ^#^p < 0.05. (C1)

In the beginning, we have ensured the radiosensitization of three groups cells are make sense. Clonogenic survival assay can reveal the survival score of irradiated cells veritably and is thus widely used in measuring the radio-sensitivity of cells. The higher survival fraction under 2 Gy radiation (SF2) of NC cells was 0.53, IM cells were 0.78, IC cells was 0.62 obtained from the linear-quadratic formulation (L-Q) of cell-absorbed dose survival equation [Figure 5 A1, A3]. In a multi-target single-hit model, the sensitization enhancement ratio (SER) of IM/IC was 0.46; IC/NC was 0.76 [Figure 5 A2, A4].

To further validate our hypothesis, the amount of CD133+ cells in NC was 2.39±0.244%, significantly increased to 15.73±1.93% in IM [p<0.05]. After treated with CUR, decreased to 5.51±0.70% in IC [p<0.05]. [Figure 5 B1-B4]. The wound healing assay also showed that the transferability in the IM group was enhanced, and the transferability in IC group was weakened after the intervention [p < 0.05] [Figure 5 C1-C2].

## Discussion

In this study, we found that several circRNAs and their corresponding miRNAs were related to the radiation sensitivity of NPC, and curcumin was found to restore the expression of some of these circRNAs. The circRNAs serve as natural miRNA sponges by competitively binding to miRNA response elements (MREs) and suppress their expression and function. The miRNAs in cancer are commonly involved in destabilization and degradation of mRNAs 30. This regulatory pathway may be linked to the changes in tumor stem-like properties of radiation-resistant NPC cells. Tumor stem-like cells have been shown to exist in a variety of tumors including NPC 7. The results of flow cytometry in this study have confirmed the tumor stem-like cells in CNE-2 cell line incredible increase after long term exposure to radiation, and CUR can reverse this to some extent. And that just confirmed our hypothesis, continuous exposure to radiation-induced abnormal expression of circRNAs leading to the differentiation of nasopharyngeal epithelial stem cells along with an attempt of self-renewal to replace the impaired cells. The results of GO analysis of circRNAs have shown that circRNAs have a high rate of expression in skin cancer, head and neck cancer, NPC, and embryonic stem cells.

DNA double chain damage repair is an essential mechanism of resistance of tumor cells to radiotherapy 31. DNA damage can induce DNA damage response signaling (DDR signaling) in the tumor cells. This reaction enables the cells to eliminate or reduce damage and allows to stimulate apoptotic pathways to prevent severe mutations. Studies have shown that resistance to radiotherapy is associated with DDR signaling in the tumor stem cells 32. The tumor stem-like cells have high malignant potentials and strong proliferative and transformation properties 33. Under 2 Gy radiation, the higher survival fraction (SF2) indicates stronger radio- resistivity, CUR can reduce SF2 increasing induced by radiation in IM CNE-2 cells. Also, the changes of SER in three groups showed CUR could radiosensitize CNE-2 cells in this study. In the meantime, our research found that NPC CNE-2 cells had a significantly increased rate of metastasis after acquiring resistance to radiation, while the transfer capacity of the cells was restored to a weaker magnitude after treatment with curcumin. As we all know, the direct impact and potential of NPC radioresistant had been extensively investigated and elucidated in a great number of previous studies with very consistent findings and conclusions. However, it seems almost nobody cares about the different mechanisms induced NPC radioresistant between long term and transient exposure to radiation. And does the amount of CD133+ cells changed in these two radioresistance NPC cells? How can we decrease the CD133+ cells in radiosensitive CNE-2 cells? We have taken a year to create a stable radioresistant NPC cell line and trying to figure out these concerns.^34^ The assessment of the up- and down-stream targets would be studied by CRIPSR-Cas9 and NAVI system^35^.These mechanisms could be used for gene therapy NPC radioresistance in *vivo*, animal models even clinical.

CircRNAs are a particular type of noncoding RNA molecules that lack 5′-3′ ends and poly-A tail. CircRNAs have closed ring structures that are not affected by RNA excision enzyme 36. CircRNAs have high abundance, stability, biological conservatism, and tissue specificity. The mRNA precursor is cut and the shear supply is connected to the upstream shear receptor through a splicing mechanism called back-splices. CircRNAs may enhance or inhibit carcinogenesis by inhibiting miRNAs associated with differentiation, proliferation, migration, and carcinogenic effects.

Validation of circRNAs and their corresponding miRNAs and mRNAs by bioinformatical analysis through screening demonstrated that the resistance of NPC CNE-2 cells to radiation was regulated by the change in the “hsa_circRNA_102115”-“hsa-miR-335-3p” -“IGF1R/MAPK1” network. Curcumin restored the sensitivity of NPC CNE-2 cells to radiation by regulating the “hsa_circRNA_102115”-“hsa-miR-335-3p”-“MAPK1” network. The results of the GO analysis also showed that the primary molecular functions which were involved were transcription factor activity and transcription regulator activity, while the main biological processes which were involved were regulation of nucleobase, nucleoside, nucleotide, and nucleic acid metabolism. Studies have shown that circRNAs play a vital role in the regulation of post-transcriptional gene expression 37. RNA binding proteins are involved in a variety of biological activities in the post-transcription levels, including cell proliferation, differentiation, movement, apoptosis, aging, and cell response to oxidative stress. RNA binds to these proteins to form an RNA-protein complex (RPCs), which can be used as an RNA binding protein sponge. These RPCs regulate RNA binding proteins and miRNAs and then interact with the corresponding linear RNAs 37, 38.

In this study, we found that the resistance of NPC CNE-2 cells to radiation was related to the tumor stem-like cells present in it. This might be related to the changes in the interaction network "hsa_circRNA_102115"-"hsa-miR-335-3p"-"IGF1R/MAPK1" induced by radiation. Curcumin could restore the radiation sensitivity of the NPC cells by regulating the change in expression of the "hsa_circRNA_102115"-"hsa-miR-335-3p"-"MAPK1" interaction network.

## Supplementary Material

Supplementary table.

## Abbreviations

NC: negative control
IM: irradiation model
IC: irradiation and curcumin
