# Supplementary material for: Curcumin Enhances Radiosensitization of Nasopharyngeal Carcinoma via Mediating Regulation of Tumor Stem-like Cells by a CircRNA Network
Source: J Cancer. 2020 Feb 10;11(8):2360–70. doi: 10.7150/jca.39511 (PMC7052922; doi:10.7150/jca.39511)
Supplement: Supplementary file 1 — Supplementary table. [file jcav11p2360s1.pdf]

Supplementary material

Supplementary Table S1 Primers used for qRT-PCR.

mRNA	
IGF1R-F	GAGAGGAGCAGCTAGAAGGG
IGF1R-R	CCCTTTAGTCCCCGTCACTT
FGFR1-F	ATCGAGGTGAATGGGAGCAA
FGFR1-R	CTCTTCCAGGGCTTCCAGAA
MAPK1-F	GTGACCTCAAGCCTTCCAAC
MAPK1-R	AGAATGCAGCCTACAGACCA
MAPK3-F	AAGATCAGCCCCTTCGAACA
MAPK3-R	CCATCAGGTCCTGCACAATG
SMAD2-F	CTTTGTGCAGAGCCCCAATT
SMAD2-R	CTTGTTACCGTCTGCCTTCG
CTNNB1-F	CTTACACCCACCATCCCACT
CTNNB1-R	CCTCCACAAATTGCTGCTGT
GAPDH-F	ATCATCAGCAATGCCTCCTG
GAPDH-R	ATGGACTGTGGTCATGAGTC
circRNA	
hsa_circRNA_102115-F	ATCGAGAATTGTTGCAGGGC
hsa_circRNA_102115-R	CGTCAGCCGCTCATGG
hsa_circRNA_104057-F	AGAGGCCTTACAAGTGCACT
hsa_circRNA_104057-R	CCTCTTTCGTTTCCTGTGCA
hsa_circRNA_103572-F	TGCAGGCGAAATGTGGAAAA
hsa_circRNA_103572-R	AGGTCTGTCATCACTCTGAGG
hsa_circRNA_004868-F	GAGTGAGTGGCTTAACAGTG
hsa_circRNA_004868-R	GTACCAACCTGTTCCAG
hsa_circRNA_100912-F	TGCTGCAACAATGGATTCACT
hsa_circRNA_100912-R	ATTAAGGCCAGCTGAAGGGT
hsa_circRNA_102857-F	GCTACATCCAGGAGAGAATGC
hsa_circRNA_102857-R	AAGCCAAATCACCTGTCTGC
hsa_circRNA_402801-F	GCGTGAACTATAAGGAGCCC
hsa_circRNA_402801-R	TGAGTTTCAGGTGGTGTAGTTT

miRNA		
hsa-miR-335-3p	RV-MR	GTCGTACCAGTGCAGGGTCCGAGGTATTCGCACTGGATACGACGGTCAG
	QPF	GGCCTTTTTCATTATTGCTCC
	QPR	GTGCAGGGTCCGAGGTATT
hsa-miR-19a-3p	RV-MR	GTCGTACCAGTGCAGGGTCCGAGGTATTCGCACTGGATACGACTCAGTT
	QPF	CGTGTGCAAATCTATGCAAAA
	QPR	GTGCAGGGTCCGAGGTATT
hsa-miR-544a	RV-MR	GTCGTACCAGTGCAGGGTCCGAGGTATTCGCACTGGATACGACGAACTT
	QPF	GGGATTCTGCATTTTTAGCAAG
	QPR	GTGCAGGGTCCGAGGTATT
hsa-miR-4422	RV-MR	GTCGTACCAGTGCAGGGTCCGAGGTATTCGCACTGGATACGACTGGGTA
	QPF	CAAAAGCATCAGGAAGTACCC
	QPR	GTGCAGGGTCCGAGGTATT
hsa-miR-9-5p	RV-MR	GTCGTACCAGTGCAGGGTCCGAGGTATTCGCACTGGATACGACTCATAC
	QPF	CCCGTCTTTGGTTATCTAGCTG
	QPR	GTGCAGGGTCCGAGGTATT
hsa-miR-450b-5p	RV-MR	GTCGTACCAGTGCAGGGTCCGAGGTATTCGCACTGGATACGACTATTCA
	QPF	CCCTTTTGCAATATGTTCCTG
	QPR	GTGCAGGGTCCGAGGTATT
hsa-miR-4719	RV-MR	GTCGTACCAGTGCAGGGTCCGAGGTATTCGCACTGGATACGACCCTGCA
	QPF	CGCGCGTCACAAATCTATAATA
	QPR	GTGCAGGGTCCGAGGTATT
U6	RV-MR	CGCTTCACGAATTTGCGTGTCAT
	QPF	CTCGCTTCGGCAGCACA
